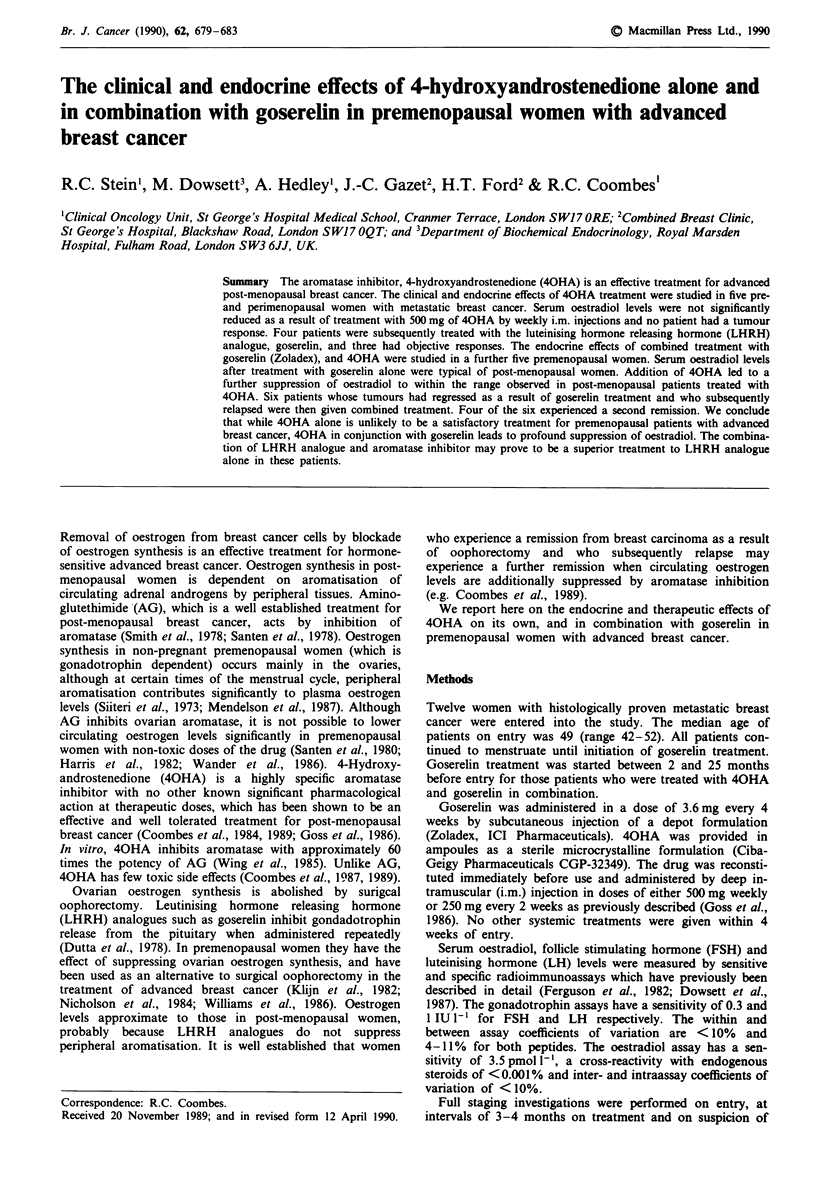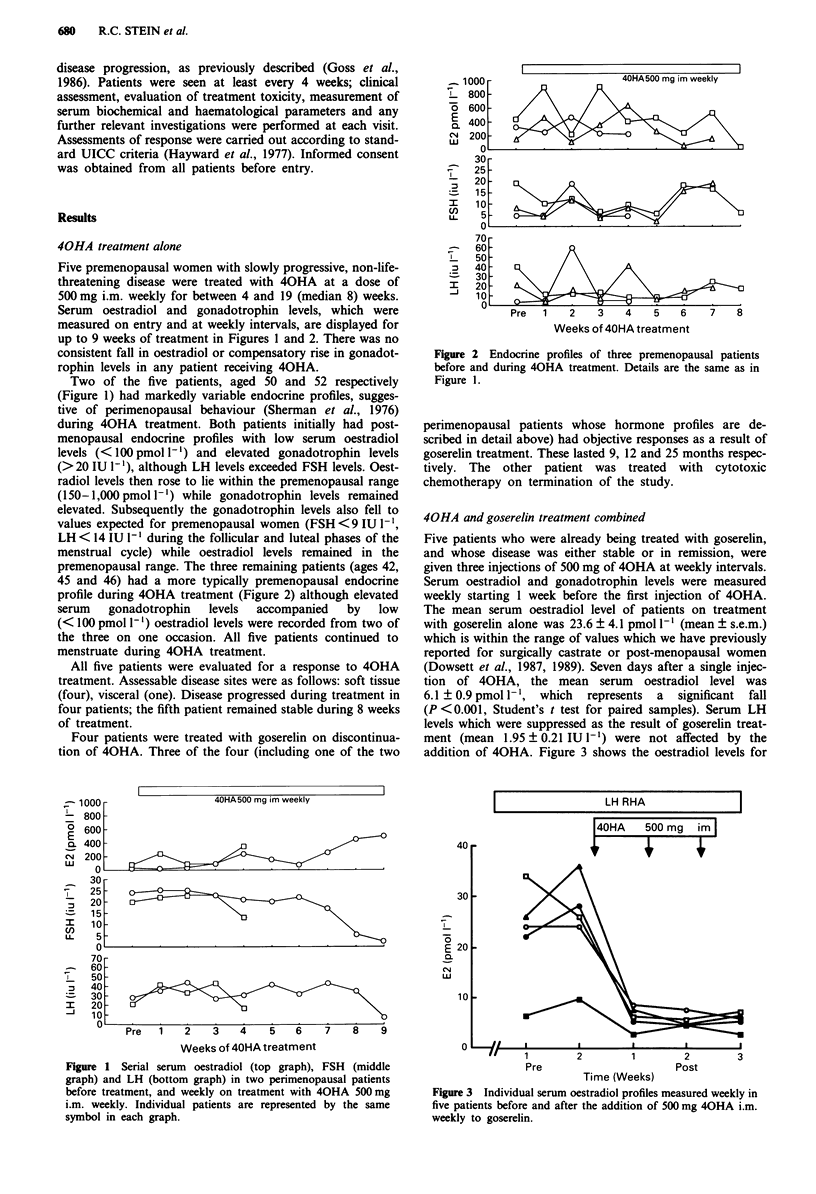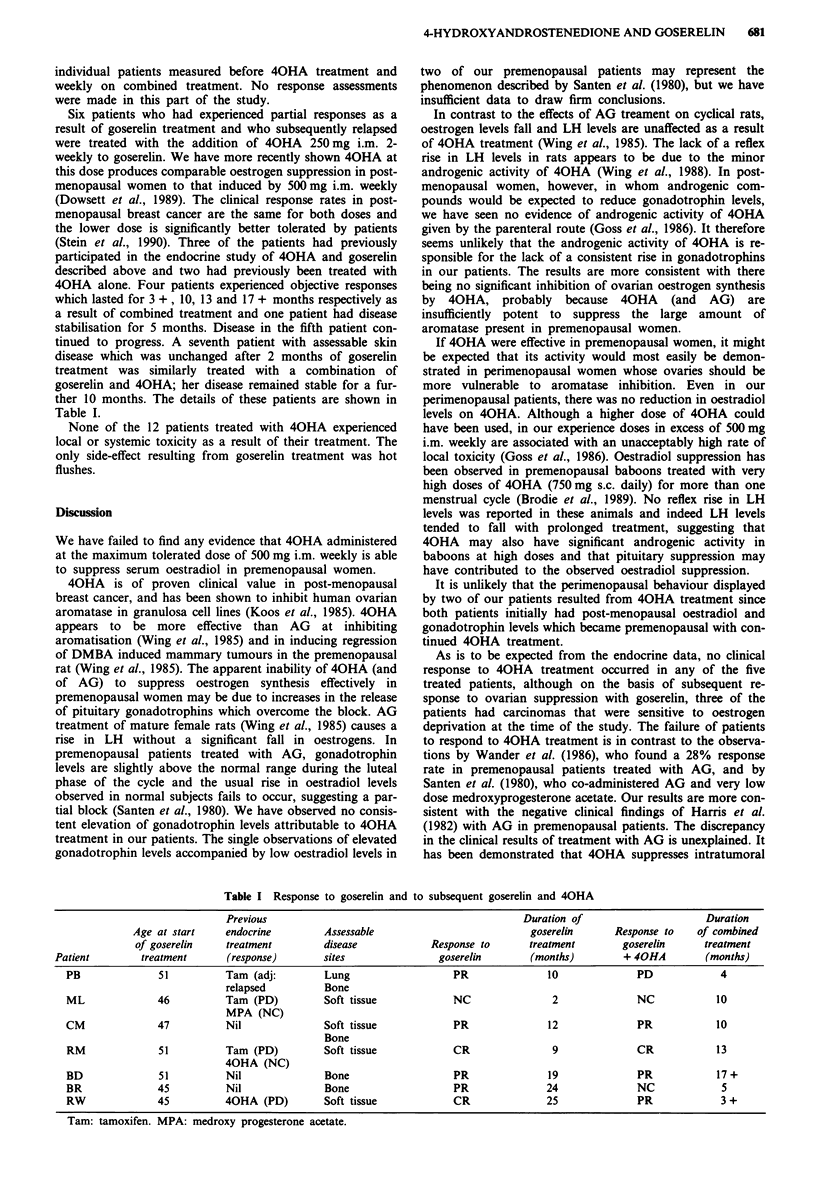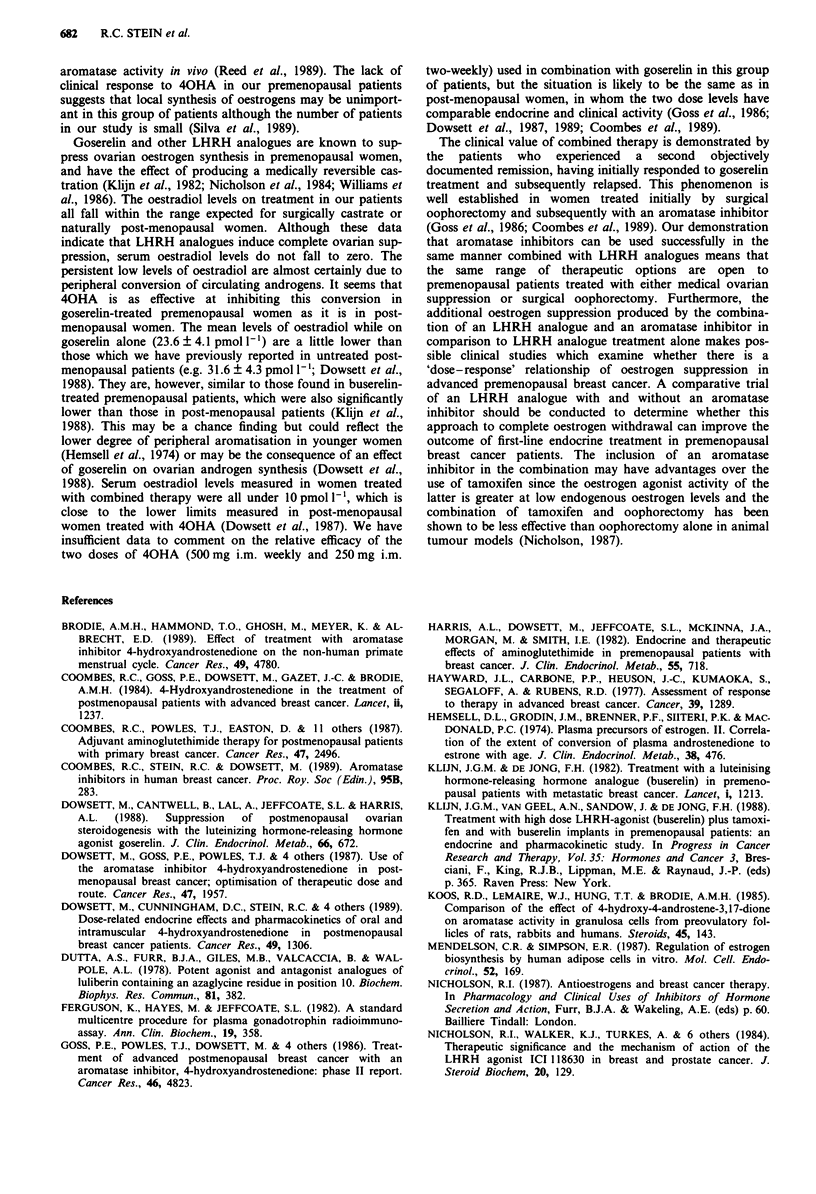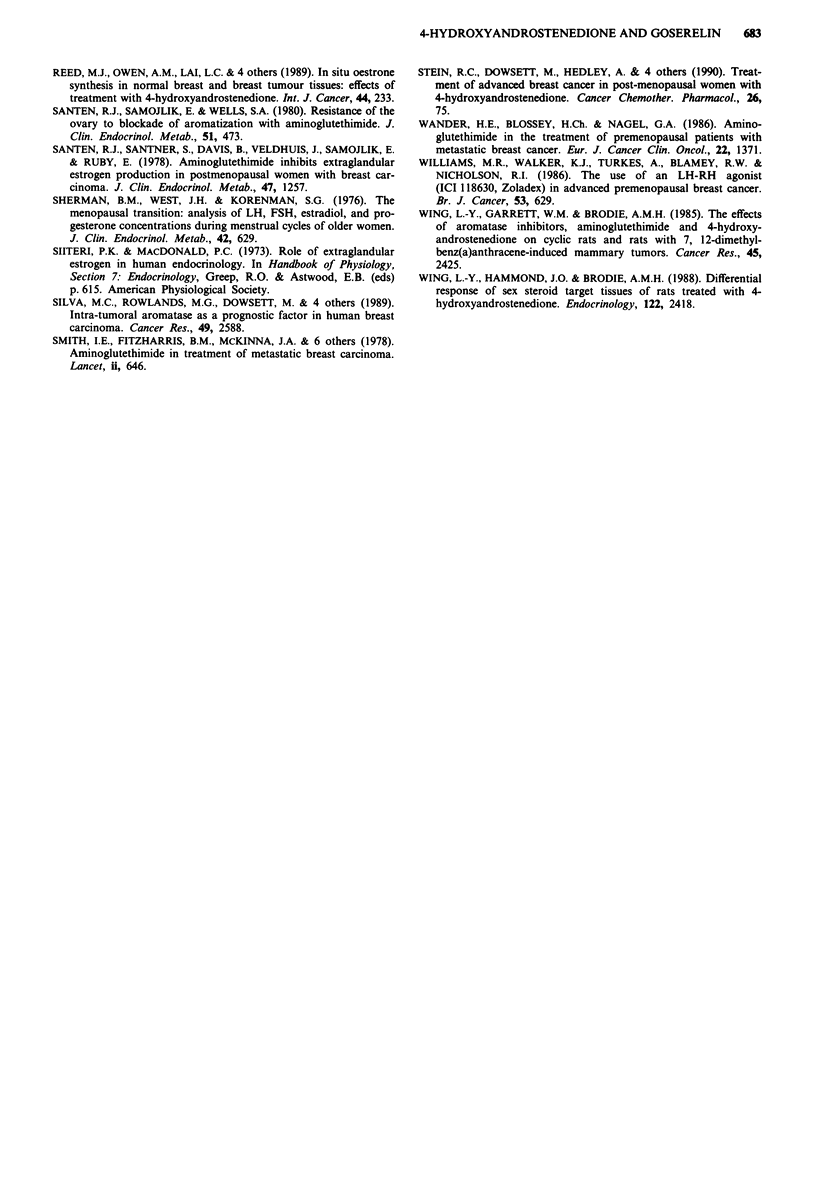# The clinical and endocrine effects of 4-hydroxyandrostenedione alone and in combination with goserelin in premenopausal women with advanced breast cancer.

**DOI:** 10.1038/bjc.1990.356

**Published:** 1990-10

**Authors:** R. C. Stein, M. Dowsett, A. Hedley, J. C. Gazet, H. T. Ford, R. C. Coombes

**Affiliations:** Clinical Oncology Unit, St George's Hospital Medical School, London, UK.

## Abstract

The aromatase inhibitor, 4-hydroxyandrostenedione (4OHA) is an effective treatment for advanced post-menopausal breast cancer. The clinical and endocrine effects of 4OHA treatment were studied in five pre- and perimenopausal women with metastatic breast cancer. Serum oestradiol levels were not significantly reduced as a result of treatment with 500 mg of 4OHA by weekly i.m. injections and no patient had a tumour response. Four patients were subsequently treated with the luteinising hormone releasing hormone (LHRH) analogue, gosereline, and three had objective responses. The endocrine effects of combined treatment with goserelin (Zoladex), and 4OHA were studied in a further five premenopausal women. Serum oestradiol levels after treatment with goserelin alone were typical of post-menopausal women. Addition of 4OHA led to a further suppression of oestradiol to within the range observed in post-menopausal patients treated with further suppression of oestradiol to within the range observed in post-menopausal patients treated with 4OHA. Six patients whose tumours had regressed as a result of goserelin treatment and who subsequently relapsed were then given combined treatment. Four of the six experienced a second remission. We conclude that while 4OHA alone is unlikely to be a satisfactory treatment for premenopausal patients with advanced breast cancer, 4OHA in conjunction with goserelin leads to profound suppression of oestradiol. The combination of LHRH analogue and aromatase inhibitor may prove to be a superior treatment to LHRH analogue alone in these patients.


Br. J. Cancer (1990), 62, 679-683 © Macmillan Press Ltd., 1990

### The clinical and endocrine effects of 4-hydroxyandrostenedione alone and in combination with goserelin in premenopausal women with advanced breast cancer

R.C. Stein[1], M. Dowsett[3], A. Hedley[1], J.-C. Gazet[2], H.T. Ford[2] & R.C. Coombes[1]

[1]*Clinical Oncology Unit, St George's Hospital Medical School, Cranmer Terrace, London SW17 0RE;* [2]*Combined Breast Clinic, St George's Hospital, Blackshaw Road, London SW17 0QT; and* [3]*Department of Biochemical Endocrinology, Royal Marsden Hospital, Fulham Road, London SW3 6JJ, UK.*

**Summary** The aromatase inhibitor, 4-hydroxyandrostenedione (4OHA) is an effective treatment for advanced post-menopausal breast cancer. The clinical and endocrine effects of 4OHA treatment were studied in five pre- and perimenopausal women with metastatic breast cancer. Serum oestradiol levels were not significantly reduced as a result of treatment with 500 mg of 4OHA by weekly i.m. injections and no patient had a tumour response. Four patients were subsequently treated with the luteinising hormone releasing hormone (LHRH) analogue, goserelin, and three had objective responses. The endocrine effects of combined treatment with goserelin (Zoladex), and 4OHA were studied in a further five premenopausal women. Serum oestradiol levels after treatment with goserelin alone were typical of post-menopausal women. Addition of 4OHA led to a further suppression of oestradiol to within the range observed in post-menopausal patients treated with 4OHA. Six patients whose tumours had regressed as a result of goserelin treatment and who subsequently relapsed were then given combined treatment. Four of the six experienced a second remission. We conclude that while 4OHA alone is unlikely to be a satisfactory treatment for premenopausal patients with advanced breast cancer, 4OHA in conjunction with goserelin leads to profound suppression of oestradiol. The combination of LHRH analogue and aromatase inhibitor may prove to be a superior treatment to LHRH analogue alone in these patients.

Removal of oestrogen from breast cancer cells by blockade of oestrogen synthesis is an effective treatment for hormone-sensitive advanced breast cancer. Oestrogen synthesis in post-menopausal women is dependent on aromatisation of circulating adrenal androgens by peripheral tissues. Aminoglutethimide (AG), which is a well established treatment for post-menopausal breast cancer, acts by inhibition of aromatase (Smith *et al.*, 1978; Santen *et al.*, 1978). Oestrogen synthesis in non-pregnant premenopausal women (which is gonadotrophin dependent) occurs mainly in the ovaries, although at certain times of the menstrual cycle, peripheral aromatisation contributes significantly to plasma oestrogen levels (Siiteri *et al.*, 1973; Mendelson *et al.*, 1987). Although AG inhibits ovarian aromatase, it is not possible to lower circulating oestrogen levels significantly in premenopausal women with non-toxic doses of the drug (Santen *et al.*, 1980; Harris *et al.*, 1982; Wander *et al.*, 1986). 4-Hydroxyandrostenedione (4OHA) is a highly specific aromatase inhibitor with no other known significant pharmacological action at therapeutic doses, which has been shown to be an effective and well tolerated treatment for post-menopausal breast cancer (Coombes *et al.*, 1984, 1989; Goss *et al.*, 1986). *In vitro*, 4OHA inhibits aromatase with approximately 60 times the potency of AG (Wing *et al.*, 1985). Unlike AG, 4OHA has few toxic side effects (Coombes *et al.*, 1987, 1989).

Ovarian oestrogen synthesis is abolished by surigcal oophorectomy. Leutinising hormone releasing hormone (LHRH) analogues such as goserelin inhibit gondadotrophin release from the pituitary when administered repeatedly (Dutta *et al.*, 1978). In premenopausal women they have the effect of suppressing ovarian oestrogen synthesis, and have been used as an alternative to surgical oophorectomy in the treatment of advanced breast cancer (Klijn *et al.*, 1982; Nicholson *et al.*, 1984; Williams *et al.*, 1986). Oestrogen levels approximate to those in post-menopausal women, probably because LHRH analogues do not suppress peripheral aromatisation. It is well established that women who experience a remission from breast carcinoma as a result of oophorectomy and who subsequently relapse may experience a further remission when circulating oestrogen levels are additionally suppressed by aromatase inhibition (e.g. Coombes *et al.*, 1989).

We report here on the endocrine and therapeutic effects of 4OHA on its own, and in combination with goserelin in premenopausal women with advanced breast cancer.

### Methods

Twelve women with histologically proven metastatic breast cancer were entered into the study. The median age of patients on entry was 49 (range 42-52). All patients continued to menstruate until initiation of goserelin treatment. Goserelin treatment was started between 2 and 25 months before entry for those patients who were treated with 4OHA and goserelin in combination.

Goserelin was administered in a dose of 3.6 mg every 4 weeks by subcutaneous injection of a depot formulation (Zoladex, ICI Pharmaceuticals). 4OHA was provided in ampoules as a sterile microcrystalline formulation (Ciba-Geigy Pharmaceuticals CGP-32349). The drug was reconstituted immediately before use and administered by deep intramuscular (i.m.) injection in doses of either 500 mg weekly or 250 mg every 2 weeks as previously described (Goss *et al.*, 1986). No other systemic treatments were given within 4 weeks of entry.

Serum oestradiol, follicle stimulating hormone (FSH) and luteinising hormone (LH) levels were measured by sensitive and specific radioimmunoassays which have previously been described in detail (Ferguson *et al.*, 1982; Dowsett *et al.*, 1987). The gonadotrophin assays have a sensitivity of 0.3 and $1\ IU\ l^{-1}$ for FSH and LH respectively. The within and between assay coefficients of variation are $<10\%$ and 4-11% for both peptides. The oestradiol assay has a sensitivity of $3.5\ pmol\ l^{-1}$, a cross-reactivity with endogenous steroids of $<0.001\%$ and inter- and intraassay coefficients of variation of $<10\%$.

Full staging investigations were performed on entry, at intervals of 3-4 months on treatment and on suspicion of

Correspondence: R.C. Coombes.
Received 20 November 1989; and in revised form 12 April 1990.

disease progression, as previously described (Goss et al., 1986). Patients were seen at least every 4 weeks; clinical assessment, evaluation of treatment toxicity, measurement of serum biochemical and haematological parameters and any further relevant investigations were performed at each visit. Assessments of response were carried out according to standard UICC criteria (Hayward et al., 1977). Informed consent was obtained from all patients before entry.

### Results

### *4OHA treatment alone*

Five premenopausal women with slowly progressive, non-life-threatening disease were treated with 4OHA at a dose of 500 mg i.m. weekly for between 4 and 19 (median 8) weeks. Serum oestradiol and gonadotrophin levels, which were measured on entry and at weekly intervals, are displayed for up to 9 weeks of treatment in Figures 1 and 2. There was no consistent fall in oestradiol or compensatory rise in gonadotrophin levels in any patient receiving 4OHA.

Two of the five patients, aged 50 and 52 respectively (Figure 1) had markedly variable endocrine profiles, suggestive of perimenopausal behaviour (Sherman et al., 1976) during 4OHA treatment. Both patients initially had post-menopausal endocrine profiles with low serum oestradiol levels (< 100 pmol $l^{-1}$) and elevated gonadotrophin levels (> 20 IU $l^{-1}$), although LH levels exceeded FSH levels. Oestradiol levels then rose to lie within the premenopausal range (150–1,000 pmol $l^{-1}$) while gonadotrophin levels remained elevated. Subsequently the gonadotrophin levels also fell to values expected for premenopausal women (FSH < 9 IU $l^{-1}$, LH < 14 IU $l^{-1}$ during the follicular and luteal phases of the menstrual cycle) while oestradiol levels remained in the premenopausal range. The three remaining patients (ages 42, 45 and 46) had a more typically premenopausal endocrine profile during 4OHA treatment (Figure 2) although elevated serum gonadotrophin levels accompanied by low (< 100 pmol $l^{-1}$) oestradiol levels were recorded from two of the three on one occasion. All five patients continued to menstruate during 4OHA treatment.

All five patients were evaluated for a response to 4OHA treatment. Assessable disease sites were as follows: soft tissue (four), visceral (one). Disease progressed during treatment in four patients; the fifth patient remained stable during 8 weeks of treatment.

Four patients were treated with goserelin on discontinuation of 4OHA. Three of the four (including one of the two perimenopausal patients whose hormone profiles are described in detail above) had objective responses as a result of goserelin treatment. These lasted 9, 12 and 25 months respectively. The other patient was treated with cytotoxic chemotherapy on termination of the study.

**Figure 2** Endocrine profiles of three premenopausal patients before and during 4OHA treatment. Details are the same as in Figure 1.

### *4OHA and goserelin treatment combined*

Five patients who were already being treated with goserelin, and whose disease was either stable or in remission, were given three injections of 500 mg of 4OHA at weekly intervals. Serum oestradiol and gonadotrophin levels were measured weekly starting 1 week before the first injection of 4OHA. The mean serum oestradiol level of patients on treatment with goserelin alone was 23.6 ± 4.1 pmol $l^{-1}$ (mean ± s.e.m.) which is within the range of values which we have previously reported for surgically castrate or post-menopausal women (Dowsett et al., 1987, 1989). Seven days after a single injection of 4OHA, the mean serum oestradiol level was 6.1 ± 0.9 pmol $l^{-1}$, which represents a significant fall ($P < 0.001$, Student's $t$ test for paired samples). Serum LH levels which were suppressed as the result of goserelin treatment (mean 1.95 ± 0.21 IU $l^{-1}$) were not affected by the addition of 4OHA. Figure 3 shows the oestradiol levels for

**Figure 1** Serial serum oestradiol (top graph), FSH (middle graph) and LH (bottom graph) in two perimenopausal patients before treatment, and weekly on treatment with 4OHA 500 mg i.m. weekly. Individual patients are represented by the same symbol in each graph.

**Figure 3** Individual serum oestradiol profiles measured weekly in five patients before and after the addition of 500 mg 4OHA i.m. weekly to goserelin.

individual patients measured before 4OHA treatment and weekly on combined treatment. No response assessments were made in this part of the study.

Six patients who had experienced partial responses as a result of goserelin treatment and who subsequently relapsed were treated with the addition of 4OHA 250 mg i.m. 2-weekly to goserelin. We have more recently shown 4OHA at this dose produces comparable oestrogen suppression in post-menopausal women to that induced by 500 mg i.m. weekly (Dowsett *et al.*, 1989). The clinical response rates in post-menopausal breast cancer are the same for both doses and the lower dose is significantly better tolerated by patients (Stein *et al.*, 1990). Three of the patients had previously participated in the endocrine study of 4OHA and goserelin described above and two had previously been treated with 4OHA alone. Four patients experienced objective responses which lasted for 3 +, 10, 13 and 17 + months respectively as a result of combined treatment and one patient had disease stabilisation for 5 months. Disease in the fifth patient continued to progress. A seventh patient with assessable skin disease which was unchanged after 2 months of goserelin treatment was similarly treated with a combination of goserelin and 4OHA; her disease remained stable for a further 10 months. The details of these patients are shown in Table I.

None of the 12 patients treated with 4OHA experienced local or systemic toxicity as a result of their treatment. The only side-effect resulting from goserelin treatment was hot flushes.

### Discussion

We have failed to find any evidence that 4OHA administered at the maximum tolerated dose of 500 mg i.m. weekly is able to suppress serum oestradiol in premenopausal women.

4OHA is of proven clinical value in post-menopausal breast cancer, and has been shown to inhibit human ovarian aromatase in granulosa cell lines (Koos *et al.*, 1985). 4OHA appears to be more effective than AG at inhibiting aromatisation (Wing *et al.*, 1985) and in inducing regression of DMBA induced mammary tumours in the premenopausal rat (Wing *et al.*, 1985). The apparent inability of 4OHA (and of AG) to suppress oestrogen synthesis effectively in premenopausal women may be due to increases in the release of pituitary gonadotrophins which overcome the block. AG treatment of mature female rats (Wing *et al.*, 1985) causes a rise in LH without a significant fall in oestrogens. In premenopausal patients treated with AG, gonadotrophin levels are slightly above the normal range during the luteal phase of the cycle and the usual rise in oestradiol levels observed in normal subjects fails to occur, suggesting a partial block (Santen *et al.*, 1980). We have observed no consistent elevation of gonadotrophin levels attributable to 4OHA treatment in our patients. The single observations of elevated gonadotrophin levels accompanied by low oestradiol levels in two of our premenopausal patients may represent the phenomenon described by Santen *et al.* (1980), but we have insufficient data to draw firm conclusions.

In contrast to the effects of AG treament on cyclical rats, oestrogen levels fall and LH levels are unaffected as a result of 4OHA treatment (Wing *et al.*, 1985). The lack of a reflex rise in LH levels in rats appears to be due to the minor androgenic activity of 4OHA (Wing *et al.*, 1988). In post-menopausal women, however, in whom androgenic compounds would be expected to reduce gonadotrophin levels, we have seen no evidence of androgenic activity of 4OHA given by the parenteral route (Goss *et al.*, 1986). It therefore seems unlikely that the androgenic activity of 4OHA is responsible for the lack of a consistent rise in gonadotrophins in our patients. The results are more consistent with there being no significant inhibition of ovarian oestrogen synthesis by 4OHA, probably because 4OHA (and AG) are insufficiently potent to suppress the large amount of aromatase present in premenopausal women.

If 4OHA were effective in premenopausal women, it might be expected that its activity would most easily be demonstrated in perimenopausal women whose ovaries should be more vulnerable to aromatase inhibition. Even in our perimenopausal patients, there was no reduction in oestradiol levels on 4OHA. Although a higher dose of 4OHA could have been used, in our experience doses in excess of 500 mg i.m. weekly are associated with an unacceptably high rate of local toxicity (Goss *et al.*, 1986). Oestradiol suppression has been observed in premenopausal baboons treated with very high doses of 4OHA (750 mg s.c. daily) for more than one menstrual cycle (Brodie *et al.*, 1989). No reflex rise in LH levels was reported in these animals and indeed LH levels tended to fall with prolonged treatment, suggesting that 4OHA may also have significant androgenic activity in baboons at high doses and that pituitary suppression may have contributed to the observed oestradiol suppression.

It is unlikely that the perimenopausal behaviour displayed by two of our patients resulted from 4OHA treatment since both patients initially had post-menopausal oestradiol and gonadotrophin levels which became premenopausal with continued 4OHA treatment.

As is to be expected from the endocrine data, no clinical response to 4OHA treatment occurred in any of the five treated patients, although on the basis of subsequent response to ovarian suppression with goserelin, three of the patients had carcinomas that were sensitive to oestrogen deprivation at the time of the study. The failure of patients to respond to 4OHA treatment is in contrast to the observations by Wander *et al.* (1986), who found a 28% response rate in premenopausal patients treated with AG, and by Santen *et al.* (1980), who co-administered AG and very low dose medroxyprogesterone acetate. Our results are more consistent with the negative clinical findings of Harris *et al.* (1982) with AG in premenopausal patients. The discrepancy in the clinical results of treatment with AG is unexplained. It has been demonstrated that 4OHA suppresses intratumoral

**Table I** Response to goserelin and to subsequent goserelin and 4OHA

| *Patient* | *Age at start of goserelin treatment* | *Previous endocrine treatment (response)* | *Assessable disease sites* | *Response to goserelin* | *Duration of goserelin treatment (months)* | *Response to goserelin + 4OHA* | *Duration of combined treatment (months)* |
|---|---|---|---|---|---|---|---|
| PB | 51 | Tam (adj: relapsed | Lung Bone | PR | 10 | PD | 4 |
| ML | 46 | Tam (PD) MPA (NC) | Soft tissue | NC | 2 | NC | 10 |
| CM | 47 | Nil | Soft tissue Bone | PR | 12 | PR | 10 |
| RM | 51 | Tam (PD) 4OHA (NC) | Soft tissue | CR | 9 | CR | 13 |
| BD | 51 | Nil | Bone | PR | 19 | PR | 17 + |
| BR | 45 | Nil | Bone | PR | 24 | NC | 5 |
| RW | 45 | 4OHA (PD) | Soft tissue | CR | 25 | PR | 3 + |

Tam: tamoxifen. MPA: medroxy progesterone acetate.

aromatase activity *in vivo* (Reed *et al.*, 1989). The lack of clinical response to 4OHA in our premenopausal patients suggests that local synthesis of oestrogens may be unimportant in this group of patients although the number of patients in our study is small (Silva *et al.*, 1989).

Goserelin and other LHRH analogues are known to suppress ovarian oestrogen synthesis in premenopausal women, and have the effect of producing a medically reversible castration (Klijn *et al.*, 1982; Nicholson *et al.*, 1984; Williams *et al.*, 1986). The oestradiol levels on treatment in our patients all fall within the range expected for surgically castrate or naturally post-menopausal women. Although these data indicate that LHRH analogues induce complete ovarian suppression, serum oestradiol levels do not fall to zero. The persistent low levels of oestradiol are almost certainly due to peripheral conversion of circulating androgens. It seems that 4OHA is as effective at inhibiting this conversion in goserelin-treated premenopausal women as it is in post-menopausal women. The mean levels of oestradiol while on goserelin alone ($23.6 \pm 4.1$ pmol $l^{-1}$) are a little lower than those which we have previously reported in untreated post-menopausal patients (e.g. $31.6 \pm 4.3$ pmol $l^{-1}$; Dowsett *et al.*, 1988). They are, however, similar to those found in buserelin-treated premenopausal patients, which were also significantly lower than those in post-menopausal patients (Klijn *et al.*, 1988). This may be a chance finding but could reflect the lower degree of peripheral aromatisation in younger women (Hemsell *et al.*, 1974) or may be the consequence of an effect of goserelin on ovarian androgen synthesis (Dowsett *et al.*, 1988). Serum oestradiol levels measured in women treated with combined therapy were all under 10 pmol $l^{-1}$, which is close to the lower limits measured in post-menopausal women treated with 4OHA (Dowsett *et al.*, 1987). We have insufficient data to comment on the relative efficacy of the two doses of 4OHA (500 mg i.m. weekly and 250 mg i.m. two-weekly) used in combination with goserelin in this group of patients, but the situation is likely to be the same as in post-menopausal women, in whom the two dose levels have comparable endocrine and clinical activity (Goss *et al.*, 1986; Dowsett *et al.*, 1987, 1989; Coombes *et al.*, 1989).

The clinical value of combined therapy is demonstrated by the patients who experienced a second objectively documented remission, having initially responded to goserelin treatment and subsequently relapsed. This phenomenon is well established in women treated initially by surgical oophorectomy and subsequently with an aromatase inhibitor (Goss *et al.*, 1986; Coombes *et al.*, 1989). Our demonstration that aromatase inhibitors can be used successfully in the same manner combined with LHRH analogues means that the same range of therapeutic options are open to premenopausal patients treated with either medical ovarian suppression or surgical oophorectomy. Furthermore, the additional oestrogen suppression produced by the combination of an LHRH analogue and an aromatase inhibitor in comparison to LHRH analogue treatment alone makes possible clinical studies which examine whether there is a 'dose–response' relationship of oestrogen suppression in advanced premenopausal breast cancer. A comparative trial of an LHRH analogue with and without an aromatase inhibitor should be conducted to determine whether this approach to complete oestrogen withdrawal can improve the outcome of first-line endocrine treatment in premenopausal breast cancer patients. The inclusion of an aromatase inhibitor in the combination may have advantages over the use of tamoxifen since the oestrogen agonist activity of the latter is greater at low endogenous oestrogen levels and the combination of tamoxifen and oophorectomy has been shown to be less effective than oophorectomy alone in animal tumour models (Nicholson, 1987).

### References

BRODIE, A.M.H., HAMMOND, T.O., GHOSH, M., MEYER, K. & ALBRECHT, E.D. (1989). Effect of treatment with aromatase inhibitor 4-hydroxyandrostenedione on the non-human primate menstrual cycle. *Cancer Res.*, **49**, 4780.

COOMBES, R.C., GOSS, P.E., DOWSETT, M., GAZET, J.-C. & BRODIE, A.M.H. (1984). 4-Hydroxyandrostenedione in the treatment of postmenopausal patients with advanced breast cancer. *Lancet*, **ii**, 1237.

COOMBES, R.C., POWLES, T.J., EASTON, D. & 11 others (1987). Adjuvant aminoglutethimide therapy for postmenopausal patients with primary breast cancer. *Cancer Res.*, **47**, 2496.

COOMBES, R.C., STEIN, R.C. & DOWSETT, M. (1989). Aromatase inhibitors in human breast cancer. *Proc. Roy. Soc (Edin.)*, **95B**, 283.

DOWSETT, M., CANTWELL, B., LAL, A., JEFFCOATE, S.L. & HARRIS, A.L. (1988). Suppression of postmenopausal ovarian steroidogenesis with the luteinizing hormone-releasing hormone agonist goserelin. *J. Clin. Endocrinol. Metab.*, **66**, 672.

DOWSETT, M., GOSS, P.E., POWLES, T.J. & 4 others (1987). Use of the aromatase inhibitor 4-hydroxyandrostenedione in postmenopausal breast cancer; optimisation of therapeutic dose and route. *Cancer Res.*, **47**, 1957.

DOWSETT, M., CUNNINGHAM, D.C., STEIN, R.C. & 4 others (1989). Dose-related endocrine effects and pharmacokinetics of oral and intramuscular 4-hydroxyandrostenedione in postmenopausal breast cancer patients. *Cancer Res.*, **49**, 1306.

DUTTA, A.S., FURR, B.J.A., GILES, M.B., VALCACCIA, B. & WALPOLE, A.L. (1978). Potent agonist and antagonist analogues of luliberin containing an azaglycine residue in position 10. *Biochem. Biophys. Res. Commun.*, **81**, 382.

FERGUSON, K., HAYES, M. & JEFFCOATE, S.L. (1982). A standard multicentre procedure for plasma gonadotrophin radioimmunoassay. *Ann. Clin. Biochem.*, **19**, 358.

GOSS, P.E., POWLES, T.J., DOWSETT, M. & 4 others (1986). Treatment of advanced postmenopausal breast cancer with an aromatase inhibitor, 4-hydroxyandrostenedione: phase II report. *Cancer Res.*, **46**, 4823.

HARRIS, A.L., DOWSETT, M., JEFFCOATE, S.L., McKINNA, J.A., MORGAN, M. & SMITH, I.E. (1982). Endocrine and therapeutic effects of aminoglutethimide in premenopausal patients with breast cancer. *J. Clin. Endocrinol. Metab.*, **55**, 718.

HAYWARD, J.L., CARBONE, P.P., HEUSON, J.-C., KUMAOKA, S., SEGALOFF, A. & RUBENS, R.D. (1977). Assessment of response to therapy in advanced breast cancer. *Cancer*, **39**, 1289.

HEMSELL, D.L., GRODIN, J.M., BRENNER, P.F., SIITERI, P.K. & MACDONALD, P.C. (1974). Plasma precursors of estrogen. II. Correlation of the extent of conversion of plasma androstenedione to estrone with age. *J. Clin. Endocrinol. Metab.*, **38**, 476.

KLIJN, J.G.M. & DE JONG, F.H. (1982). Treatment with a luteinising hormone-releasing hormone analogue (buserelin) in premenopausal patients with metastatic breast cancer. *Lancet*, **i**, 1213.

KLIJN, J.G.M., VAN GEEL, A.N., SANDOW, J. & DE JONG, F.H. (1988). Treatment with high dose LHRH-agonist (buserelin) plus tamoxifen and with buserelin implants in premenopausal patients: an endocrine and pharmacokinetic study. In *Progress in Cancer Research and Therapy, Vol. 35: Hormones and Cancer 3*, Bresciani, F., King, R.J.B., Lippman, M.E. & Raynaud, J.-P. (eds) p. 365. Raven Press: New York.

KOOS, R.D., LEMAIRE, W.J., HUNG, T.T. & BRODIE, A.M.H. (1985). Comparison of the effect of 4-hydroxy-4-androstene-3,17-dione on aromatase activity in granulosa cells from preovulatory follicles of rats, rabbits and humans. *Steroids*, **45**, 143.

MENDELSON, C.R. & SIMPSON, E.R. (1987). Regulation of estrogen biosynthesis by human adipose cells in vitro. *Mol. Cell. Endocrinol.*, **52**, 169.

NICHOLSON, R.I. (1987). Antioestrogens and breast cancer therapy. In *Pharmacology and Clinical Uses of Inhibitors of Hormone Secretion and Action*, Furr, B.J.A. & Wakeling, A.E. (eds) p. 60. Bailliere Tindall: London.

NICHOLSON, R.I., WALKER, K.J., TURKES, A. & 6 others (1984). Therapeutic significance and the mechanism of action of the LHRH agonist ICI 118630 in breast and prostate cancer. *J. Steroid Biochem*, **20**, 129.

REED, M.J., OWEN, A.M., LAI, L.C. & 4 others (1989). In situ oestrone synthesis in normal breast and breast tumour tissues: effects of treatment with 4-hydroxyandrostenedione. *Int. J. Cancer*, **44**, 233.

SANTEN, R.J., SAMOJLIK, E. & WELLS, S.A. (1980). Resistance of the ovary to blockade of aromatization with aminoglutethimide. *J. Clin. Endocrinol. Metab.*, **51**, 473.

SANTEN, R.J., SANTNER, S., DAVIS, B., VELDHUIS, J., SAMOJLIK, E. & RUBY, E. (1978). Aminoglutethimide inhibits extraglandular estrogen production in postmenopausal women with breast carcinoma. *J. Clin. Endocrinol. Metab.*, **47**, 1257.

SHERMAN, B.M., WEST, J.H. & KORENMAN, S.G. (1976). The menopausal transition: analysis of LH, FSH, estradiol, and progesterone concentrations during menstrual cycles of older women. *J. Clin. Endocrinol. Metab.*, **42**, 629.

SIITERI, P.K. & MACDONALD, P.C. (1973). Role of extraglandular estrogen in human endocrinology. In *Handbook of Physiology, Section 7: Endocrinology*, Greep, R.O. & Astwood, E.B. (eds) p. 615. American Physiological Society.

SILVA, M.C., ROWLANDS, M.G., DOWSETT, M. & 4 others (1989). Intra-tumoral aromatase as a prognostic factor in human breast carcinoma. *Cancer Res.*, **49**, 2588.

SMITH, I.E., FITZHARRIS, B.M., MCKINNA, J.A. & 6 others (1978). Aminoglutethimide in treatment of metastatic breast carcinoma. *Lancet*, **ii**, 646.

STEIN, R.C., DOWSETT, M., HEDLEY, A. & 4 others (1990). Treatment of advanced breast cancer in post-menopausal women with 4-hydroxyandrostenedione. *Cancer Chemother. Pharmacol.*, **26**, 75.

WANDER, H.E., BLOSSEY, H.Ch. & NAGEL, G.A. (1986). Aminoglutethimide in the treatment of premenopausal patients with metastatic breast cancer. *Eur. J. Cancer Clin. Oncol.*, **22**, 1371.

WILLIAMS, M.R., WALKER, K.J., TURKES, A., BLAMEY, R.W. & NICHOLSON, R.I. (1986). The use of an LH-RH agonist (ICI 118630, Zoladex) in advanced premenopausal breast cancer. *Br. J. Cancer*, **53**, 629.

WING, L.-Y., GARRETT, W.M. & BRODIE, A.M.H. (1985). The effects of aromatase inhibitors, aminoglutethimide and 4-hydroxyandrostenedione on cyclic rats and rats with 7, 12-dimethylbenz(a)anthracene-induced mammary tumors. *Cancer Res.*, **45**, 2425.

WING, L.-Y., HAMMOND, J.O. & BRODIE, A.M.H. (1988). Differential response of sex steroid target tissues of rats treated with 4-hydroxyandrostenedione. *Endocrinology*, **122**, 2418.